# Mouse vision as a gateway for understanding how experience shapes neural circuits

**DOI:** 10.3389/fncir.2014.00123

**Published:** 2014-10-02

**Authors:** Nicholas J. Priebe, Aaron W. McGee

**Affiliations:** ^1^Section of Neurobiology, School of Biological Sciences, University of Texas at AustinAustin, TX, USA; ^2^Developmental Neuroscience Program, Saban Research Institute, Children’s Hospital of Los Angeles, Department of Pediatrics, Keck School of Medicine, University of Southern CaliforniaLos Angeles, CA, USA

**Keywords:** ocular dominance plasticity, visual cortex, binocularity, inhibition, development

## Abstract

Genetic programs controlling ontogeny drive many of the essential connectivity patterns within the brain. Yet it is activity, derived from the experience of interacting with the world, that sculpts the precise circuitry of the central nervous system. Such experience-dependent plasticity has been observed throughout the brain but has been most extensively studied in the neocortex. A prime example of this refinement of neural circuitry is found in primary visual cortex (V1), where functional connectivity changes have been observed both during development and in adulthood. The mouse visual system has become a predominant model for investigating the principles that underlie experience-dependent plasticity, given the general conservation of visual neural circuitry across mammals as well as the powerful tools and techniques recently developed for use in rodent. The genetic tractability of mice has permitted the identification of signaling pathways that translate experience-driven activity patterns into changes in circuitry. Further, the accessibility of visual cortex has allowed neural activity to be manipulated with optogenetics and observed with genetically-encoded calcium sensors. Consequently, mouse visual cortex has become one of the dominant platforms to study experience-dependent plasticity.

The establishment of normal primary visual cortex (V1) binocularity and depth perception (stereopsis) in humans depends critically on visual experience, particularly during development (McKee et al., 2003). Disrupting concordant vision between both eyes early in life generates amblyopia, a visual deficiency that cannot be explained by alterations in retinal function of the affected eye (Hubel and Wiesel, 1965; Lepard, 1975; Kiorpes et al., 1998). Amblyopia can arise due to either a difference in depth of focus between the two eyes (anisometropia) or from the eyes not properly moving in parallel (strabismus), and it is thought to occur in 1–5% of the human population (Webber and Wood, 2005). Amblyopia results in a number of deficits in spatial vision, including lower visual acuity and depth perception (Levi et al., 1979; Harwerth and Levi, 1983; McKee et al., 2003). While patching the non-affected eye is current standard of care for improving function of the affected eye, this approach is less effective after adolescence, a time in life characterized by the close of what is termed the critical period for brain circuit plasticity. For each sensory system there exists a developmental period in which experience has a remarkable role in shaping cortical connectivity and beyond which this influence is mostly lost (Simons and Land, 1987; Lendvai et al., 2000; Zhang et al., 2002; de Villers-Sidani et al., 2007; Poo and Isaacson, 2007). Understanding the mechanisms that both govern and drive experience-dependent plasticity during the critical period, as well as those that control the timing of the critical period, could provide therapeutic interventions to improve recovery from amblyopia and other neurodevelopmental disorders.

## Conservation of neural circuitry for vision

The functional convergence of right and left eye information occurs in V1; binocular integration within V1 has become the primary platform for studying experience-dependent plasticity. Normally, the information from the two eyes is combined in V1 to generate a three-dimensional representation of the visual world: because the two eyes are horizontally offset they signal distinct perspectives on the visual scene, and those distinct signals are used to compute the distances of objects in the world. The monocular signals from the two retinae leave the eye via the optic tract and cross at the optic chiasm. In mammals, the axons of neurons located in the nasal portion of the retina cross the midline in the optic chiasm and project to subcortical targets on the contralateral side via the optic tract. Neurons from the temporal portion of the retina, in contrast, project to ipsilateral subcortical targets. This specific crossing pattern ensures that animals with frontally-positioned eyes (e.g., cat, ferret, primate) will have signals from both eyes for corresponding regions of the retinae. Importantly, the projections from the contralateral and ipsilateral eyes innervate separate sections of their subcortical targets. For example, retinal ganglion cell axons that innervate the visual thalamus lateral geniculate nucleus (LGN), provide inputs to separate portions of the LGN, and thus the LGN relay cells that project to V1 are monocular. The binocularity observed in V1 is therefore primarily due to a mixing of monocular inputs from the LGN relay cells.

**Figure 1:**
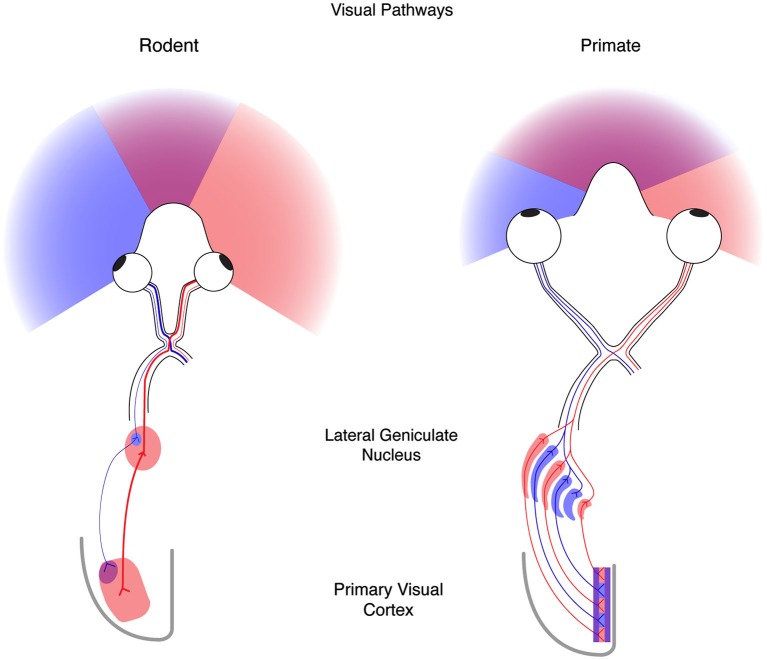
**The mouse and human visual systems share basic similarities but differ in complexity**. Right, a schematic of the rodent visual system. The eyes in the rodent are positioned laterally resulting in hemi-panoramic vision that includes a narrow central binocular zone (purple) flanked by regions of monocular vison (blue and red). Retinal ganglion cells (RGCs) with receptive fields in the binocular zone from the ipsilateral eye (blue) send a minor projection to a discrete patch in the lateral geniculate nucleus (LGN), whereas the contralateral eye (red) provides the predominant innervation to the LGN. Thalamocortical projections from these two regions converge on the binocular zone (purple) in primary visual cortex (V1). Left, a schematic of the human visual system. Forward facing eyes provide for a more expansive zone of binocular vision. Retinal ganglion cells from the two eyes send similar projections to the LGN that are distributed to eye-specific laminae. Thalamocortical projections similarly converge on V1 (purple) but also maintain evident regions of enrichment termed ocular dominance columns.

Because experience drives similar changes in both the functional response properties of cortical neurons and the anatomical projections to visual cortex from the thalamus across mammals (Antonini and Stryker, 1993; Antonini et al., 1999), the ease of accessibility and genetics, as well as the compendium of available tools, techniques, and resources for mouse has led to it becoming a standard system to investigate both the governing principles and mechanisms necessary for activity-dependent plasticity. That said, while the mouse has a number of advantages as a model system, it is important to note that in addition to similarities, there also exist large differences between rodents and other mammals that have been studied previously. One of the primary differences is the positioning of the two eyes (Figure 1). In the rodent the eyes are positioned laterally, in contrast to the frontal location of human eyes. This hemi-panoramic vision has consequences for studying cortical binocularity, as the visual world seen by both eyes in front of the mouse is small, covering only the central 50° (Drager, 1978), compared to 135° in man. Therefore, much of the mouse visual system is devoted to monocular—rather than binocular—vision. This difference in eye placement is evident at the optic chiasm: in man approximately 45% of retinal ganglion cell axons project to the ipsilateral LGN, whereas in the mouse only 4% of retinal ganglion cell axons project to the ipsilateral LGN (Dräger, 1974; Godement et al., 1984). Additionally, the anatomical organization of the LGN is distinct in the human and mouse (Figure 1). The human LGN contains multiple segregated eye-specific laminae, whereas the mouse LGN is not laminar but dominated by the contralateral eye with only a small ipsilateral patch (Dräger, 1974). Finally, the functional and anatomical organization of eye-specific signals in primary visual cortex (V1) differs between primates and mice. In primates, V1 is well characterized by a regular columnar organization for ocular dominance (OD) (Hubel and Wiesel, 1977; Adams et al., 2007). V1 neurons across cortical layers share preference for one eye over the other eye, and this ocular preference changes gradually at regular intervals across the surface of cortex. Because of this organization, primate V1 neurons near one another share functional selectivity. In mice, however, no such columnar organization has been observed, and V1 neurons near one another have little functional relationship to each other (Gordon et al., 1996; Antonini et al., 1999).

Despite these differences in functional architecture across mammals, visual experience sculpts the selectivity of neurons in all mammals examined to date. The effects of activity on neural circuitry are particularly pronounced within the developmental critical period. During the critical period, the functional response properties of neurons, particularly OD, may be manipulated by perturbing the incoming signals from the periphery. Changes resulting from such manipulation are durable, generally persisting through adulthood. Hubel and Wiesel demonstrated OD plasticity in cat by occluding one eye (monocular deprivation, MD) (Wiesel and Hubel, 1963) or disrupting the alignment of the two eyes (strabismus) (Hubel and Wiesel, 1965) during the developmental critical period. After MD, V1 neurons responded strongly to the open eye and weakly to the closed eye; after strabismus, V1 neurons were far less binocular than in normal animals. This decrease in binocularity arises in part from the disruption of normal synaptic integration of binocular inputs by simple cells in visual cortex (Scholl et al., 2013b). In concert with these functional changes, anatomical correlates of experience-dependent plasticity have also been observed. The LGN relay cells that provide inputs to V1 neurons undergo a period of refinement during development in the cat and the primate (Rakic, 1976; Hubel et al., 1977; LeVay et al., 1978; Löwel, 1994). Initially, the right and left eye thalamocortical projections intermix in layer IV, but over the course of development these projections become increasingly patchy and periodic.

These same patterns of activity-dependent changes during the critical period have not only been observed in mouse V1, but in all mammals in which they have been tested (e.g., rabbit: (Van Sluyters and Stewart, 1974), rat: (Maffei et al., 1992) cat: (Wiesel and Hubel, 1963) sheep: (Martin et al., 1979) hamster: (Emerson et al., 1982) macaque: (Hubel et al., 1977) marmoset: (DeBruyn and Casagrande, 1981)). Both MD and strabismus generate changes in the functional response properties of V1 neurons, causing V1 neurons to be more sensitive to the open eye in MD (Wiesel and Hubel, 1963), and less binocular following disruptions of simultaneous patterned activity from the two eyes (Hubel and Wiesel, 1965; Gordon and Stryker, 1996). Monocular deprivation also causes anatomical shifts in the thalamocortical projection, enhancing the growth of the thalamocortical axonal arbors associated with the open eye (Antonini et al., 1999).

## Genetic dissection of OD plasticity

**Figure 2:**
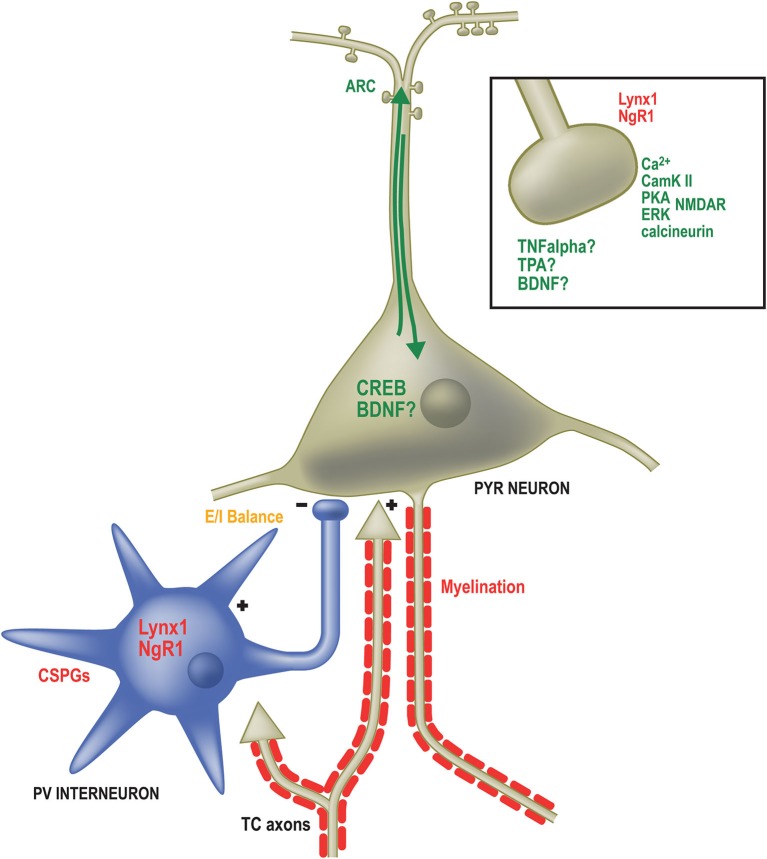
**Factors governing the expression and duration of OD plasticity operate in numerous subcellular locations**. Genes required for OD plasticity (green text) are present both at sites of synaptic contact as well as the somatodendritic compartment. Calcium signaling (Ca2+) through the NMDA receptor (NMDAR) in excitatory pyramidal (PYR) neurons activates several proteins required for OD plasticity including Calmodulin-dependent protein Kinase 2a (CamKII), Protein Kinase A (PKA), Extracellular signal-Regulated Kinase (ERK) and the phosphatase calcineurin. Likewise, Tumor Necrosis Factor alpha (TNFα), Tissue plasminogen activator (TPA) and Brain-Derived Neurotrophic Factor (BDNF) are all required for OD plasticity and may function at excitatory synapses, such as those on dendritic spines (boxed inset). Proteins restricting OD plasticity (red text) to the critical period may also function at synapses, including Nogo Receptor 1 and Lynx1. Calcium-dependent signaling proteins result in the activation of the activity-dependent transcription factor calcium/cyclic AMP binding element (CREB) as well as the immediate early gene activity-regulated cytoskeletal associated protein (ARC). Several extracellular factors are required to close the critical period and inhibit further OD plasticity. These include Chondroitin Sulfate Proteoglycans (CSPGs) that surround Parvalbumin-positive inhibitory neurons and inhibitors associated with myelin membranes. The proper balance of excitatory and inhibitory neurotransmission (E/I balance) is essential both for opening and potentially closing the critical period (orange text). Multiple approaches (not shown) that affect E/I balance affect OD plasticity.

Many specific genes have been identified as necessary for OD plasticity in mice (Figure 2). The products of such genes are known to operate at different locations within the neuron, from components of the postsynaptic density (Taha and Stryker, 2002; Taha et al., 2002; Sawtell et al., 2003; Ranson et al., 2013) to transcription factors in the nucleus (Pham et al., 1999; Mower et al., 2002) and proteins redistributed to the dendritic compartment that regulate protein stability and turnover (Tagawa et al., 2005; McCurry et al., 2010; Shepherd and Bear, 2011). These genes can be broadly categorized into two groups: (1) necessary pieces of the neural machinery to drive changes in the strength of synaptic connections; and (2) controllers of when and how much plasticity is induced.

The genes required for OD plasticity overlap with those that have been implicated in other forms of plasticity, particularly long-term potentiation (LTP) and long-term depression (LTD) (but see Rao et al., 2004). A critical synaptic factor that appears to be the first step in OD plasticity is activity at the N-methyl-D-aspartate (NMDA) receptor, which is required for synaptic plasticity and OD plasticity (Bear et al., 1990; Sawtell et al., 2003). Because the NMDA receptor (NMDAR) is voltage-gated, opening only when the neuron is already depolarized, it signals the coincident activation of incoming synaptic inputs and the activation of the neuron itself. N-methyl-D-aspartate channels are permeable to sodium, potassium and, importantly, allow the influx of calcium. It is this calcium influx that initiates the signaling cascade that eventually leads to changes in synaptic weight and that is required for normal OD plasticity.

Indeed, the calcium influx triggers a number of molecular pathways required for OD plasticity. It has been demonstrated previously that disrupting the interaction between incoming calcium and CaMKII (Taha et al., 2002), cAMP (Beaver et al., 2001; Fischer et al., 2004) or calcineurin (Yang et al., 2005) interferes with OD plasticity during the critical period. These initial calcium-driven signals lead directly or indirectly, through additional kinases such as Extracellular signal-regulated kinase (ERK; Di Cristo et al., 2001), to the activation of activity-dependent regulators of gene expression, including the calcium/cyclic AMP binding element (CREB; Pham et al., 1999, 2001). Thus, perturbing the calcium signaling pathway by weakening or eliminating a step in the cascade diminishes both synaptic plasticity and OD plasticity, providing strong evidence that synaptic modifications are a central and necessary component for the functional changes in selectivity of neurons in V1 during the critical period (Silva, 2003; Taha and Stryker, 2005).

## Genetic and circuit regulation of the critical period

In parallel with the molecular signals necessary to drive plasticity, an additional set of genes governs the timing of the critical period. While the ecological benefit of constraining plasticity to a narrow time window (P20–P32 in mice) is unclear, the conditions required for plasticity are now being uncovered. Opening the critical period requires a discrete maturation of inhibitory cortical circuitry (Levelt and Hübener, 2012). The differentiation of inhibitory neurons expressing the calcium binding protein parvalbumin (PV) precedes the onset of the critical period (Huang et al., 1999), and it has been demonstrated that OD plasticity may be induced earlier in mouse V1 by artificially increasing inhibition (Fagiolini and Hensch, 2000; Iwai et al., 2003). Indeed, increasing levels of Brain-Derived Neurotrophic Factor (BDNF), which may accelerate the maturation of inhibitory circuitry, drive a precious critical period for OD plasticity in mouse V1 (Hanover et al., 1999; Huang et al., 1999). Reducing the amount of GABA_A_ mediated inhibition in cortex, either by deleting GAD 65 (glutamic acid decarboxylase 65 kD), an enzyme required for synthesis of the inhibitory neurotransmitter GABA, or deleting the gene NARP, a pentraxin molecule required for normal excitatory drive onto inhibitory neurons during development, prevents opening of the critical period (Fagiolini and Hensch, 2000; Gu et al., 2013). Another method to delay inhibitory neuron development, and thus the critical period, is to dark-rear animals (Huang et al., 1999). Only once those animals are moved into normal lighting conditions does the critical period open. Thus, the amount of cortical inhibition, particularly inhibition mediated by PV interneurons, appears to be an essential factor in controlling the opening of the critical period for OD plasticity.

Extracellular signals play a critical role at the closure of the critical period. For example, the distribution of perineuronal nets (PNNs), which contain chondroitin sulfate proteoglycans (CSPGs) that are components of the extracellular matrix that inhibit axonal growth, plateaus at the end of the critical period (Pizzorusso et al., 2002). The distribution of myelination in visual cortex also plateaus as the critical period closes (McGee et al., 2005) and intracortical synaptogenesis begins to decline (Morales et al., 2002). Two genes related to these alterations to the extracellular environment of visual cortex are required to close the critical period. Nogo receptor 1 (NgR1) is a neuronal receptor both for CSPGs as well as several inhibitors of neurite outgrowth associated with myelin membranes (McGee and Strittmatter, 2003; Dickendesher et al., 2012). Mice that lack NgR1 continue to display OD critical period plasticity into adulthood (McGee et al., 2005). The cartilage link protein (Crtl1) also plays an essential role in closing the critical period for OD plasticity. CRT1 is a neuronal product that triggers the formation of the PNNs (Carulli et al., 2010). Normally CRT1 is upregulated in V1 as the critical period closes; mice lacking Crtl1 retain OD plasticity into adulthood like the NgR1 mutant mice (Carulli et al., 2010). In addition to these two proteins that interact with the extracellular matrix, a third gene, Lynx1, an important regulator of cholinergic tone that increases at the end of the critical period. Mice lacking Lynx1 continue to display OD plasticity into adulthood, indicating that cholinergic signaling also plays a role in closing the critical period (Morishita et al., 2010).

Upon closure of the critical period, OD plasticity is attenuated but not absent in V1. Partial shifts in OD can still be detected by single-unit recordings, though these require longer periods of MD (e.g., 6+ days in adults vs. 4 days during the critical period) (Hofer et al., 2006). During the critical period, OD plasticity appears to proceed in two stages that overlap considerably: a weakening of responses to the deprived eye followed by a homeostatic strengthening of the non-deprived eye (Frenkel and Bear, 2004; Hofer et al., 2006). This latter homeostatic component of OD plasticity requires Tumor Necrosis Factor alpha (TNFα; Kaneko et al., 2008). Intriguingly, adult plasticity is primarily confined to a slow strengthening of the non-deprived eye by a distinct mechanism that is largely independent of TNFα but requires CaMKII (Ranson et al., 2012).

## Reactivating visual plasticity in the adult

One major focus of research into OD plasticity has been to understand how, and whether, plasticity may be enhanced in adults to improve recovery from neurological disorders. The first approach demonstrating that the critical period for visual plasticity could be reopened involved injecting immature astrocytes into adult cat visual cortex (Muller and Best, 1989). Several pharmacologic and environmental manipulations subsequently have been reported to restore developmental OD plasticity to the adult visual system of rats and mice. One approach has been to disrupt the extracellular signals that prevent synaptogenesis and neurite outgrowth. Injection of chondroitinase ABC degrades the CSPGs present in PNNs surrounding PV interneurons. This treatments yields modest OD plasticity (Pizzorusso et al., 2002). How loss of these PNNs affects the function of PV interneurons or impacts cortical circuitry is not yet clear. An alternative approach has been to alter the activity of inhibitory interneurons, and thus the balance between excitation and inhibition in V1. Several strategies have been employed to do this, including direct injection of immature inhibitory neurons (Southwell et al., 2010), dark exposure (He et al., 2006), administration of fluoxetine (Maya Vetencourt et al., 2008), and environmental enrichment (Sale et al., 2007). Direct reduction of overall cortical inhibition by infusing GABAa antagonists also partially restores OD plasticity (Harauzov et al., 2010). The degree to which these approaches may affect excitatory to inhibitory balance is not yet known (Morishita and Hensch, 2008).

Classic genetics, pharmacology, and environmental manipulations have revealed important aspects of both the regulation and mechanisms of OD plasticity in mouse. The combination of sophisticated tools for manipulating and measuring neuronal function in mice is now permitting the dissection of the progression of experience-dependent plasticity through the cortical circuit with greater cell-type specificity and temporal precision. For example, a recent study revealed that OD plasticity requires a decrease in inhibitory drive from a specific inhibitory cell type (Kuhlman et al., 2013). In this study, Kuhlman et al. discover with cell-attached recordings *in vivo* that an early event following MD during the critical period is a paradoxical increase in neuronal responsiveness of pyramidal (PYR) neurons in layer (L) 2/3 to visual stimulation of either eye. This disinhibition results from a decrease in excitatory drive onto L2/3 PV neurons from L4 and is only observed with MD during the critical period. Interestingly, decreasing the activity specifically of PV neurons with designer receptors exclusively activated by designer drugs (DREADDs) (Armbruster et al., 2007; Ferguson et al., 2010) in concert with MD in adult mice results in visual plasticity indistinguishable from what is observed during the critical period. These experiments are a compelling demonstration of the utility of emerging techniques available for mouse to investigate how plasticity may originate and propagate through cortical circuitry. These available genetic and molecular tools will permit experiments in the mouse that are very difficult, at a minimum, to undertake in other animal model systems.

## OD plasticity and acuity

Short periods of MD (2–4 days) during the critical period in both mouse and cat shift OD, whereas longer MD (long-term MD, LTMD, 10 or more days) results in poor acuity in the deprived eye (Giffin and Mitchell, 1978; Prusky and Douglas, 2003). LTMD throughout the critical period has been employed as a model of amblyopia in cats and rodents for decades. The effects of LTMD on acuity may stem from a combination of changes in the periphery as well as in cortical circuitry. Lid closure can cause changes in the shape of the eye (Wallman et al., 1978), potentially disrupting optics, thus creating either myopia or hyperopia in one eye (Kiorpes and Wallman, 1995). Unequal refractive error in the eyes can then lead to changes in the cortical circuitry (e.g., Kiorpes et al., 1998). One model is that loss of cortical responsiveness to the deprived eye reduces visual acuity and the subsequent close of the critical period consolidates this visual impairment. Approaches that reactivate developmental visual plasticity, particularly when any anisometropia is corrected, may therefore be expected to improve recovery from LTMD.

Several manipulations in rodents that enhance OD plasticity also improve visual acuity following LTMD (Morishita and Hensch, 2008). Treatment with chondroitinase ABC to block extracellular signals, and environmental enrichment in combination with briefly closing the previously non-deprived eye (reverse suture), restores visual acuity in the deprived eye to normal (Pizzorusso et al., 2006; Sale et al., 2007), as does dark exposure, administration of fluoxetine, and deletion of either the Lynx1 or NgR1 gene (He et al., 2006; Morishita and Hensch, 2008; Morishita et al., 2010; Stephany et al., 2014). This string of correlation has led to the model that OD plasticity and the recovery of acuity in rodents following LTMD are linked. However, genetic dissection of the requirement for NgR1 to close the critical period reveals these facets of visual plasticity are dissociable. While completely abolishing expression of NgR1 permits both OD plasticity and recovery of acuity after LTMD, restricting deletion of NgR1 to PV maintains developmental OD plasticity in the adult but is not sufficient to improve acuity after LTMD (Stephany et al., 2014). The ability to make such specific, targeted changes in protein expression illustrates the power that the mouse model can provide to our understanding of cortical neural circuitry.

## Autism and OD plasticity

It is the hope that understanding the conditions that support critical period plasticity will eventually yield therapeutic approaches for acutely reactivating developmental plasticity, aiding in the correction of amblyopia as well as the spectrum of neurologic disorders, including autism (LeBlanc and Fagiolini, 2011), brain injury (Maurer and Hensch, 2012), and perhaps even prevention of neurodegeneration. In this regard, the sensitivity of the mouse cortex to visual disruption is particularly useful for exploring how genes implicated in syndromic forms of neurodevelopmental disorders may alter the relationship between experience and neural circuit refinement in the developing brain.

For example, OD plasticity has been examined in mouse models of Fragile X syndrome (FXS; Dölen et al., 2007) and Angelman’s syndrome (Yashiro et al., 2009; Sato and Stryker, 2010). Fragile X syndrome is a leading cause of developmental mental impairment and although symptoms vary in severity and expression, characteristic deficits include reduced intellectual abilities, hyperactivity, increased seizure susceptibility, and impaired visuo-spatial processing (Pfeiffer and Huber, 2009). Mice lacking a functional gene for fragile X mental retardation 1 *(FMR1*) phenocopy some aspects of FXS and have deficits in OD plasticity. Whereas MD during the developmental critical period decreases deprived eye responses in normal (wildtype) mice, *FMR1* mutants exhibit a potentiation of open eye responses similar to the visual plasticity resident in the adult visual system (Frenkel and Bear, 2004; Dölen et al., 2007). Whether *FMR1* mutant mice are responsive to LTMD is as yet unknown. Interestingly, *FMR1* mutant mice also display an imbalance of neocortical excitation and inhibition (Gibson et al., 2008).

Angelman’s syndrome is caused by mutations that disrupt expression of ubiquitin E3 ligase (*UBE3A*), a gene sensitive to genomic imprinting (Kishino et al., 1997; Matsuura et al., 1997). Symptoms of Angelman’s syndrome include mental impairment, seizures and behavioral abnormalities (Clayton-Smith and Laan, 2003). Ubiquitin E3 ligase mutant mice do not exhibit OD plasticity with short (3-day) MD during the critical period as measured by either visually-evoked potentials or optical imaging of intrinsic signals (Yashiro et al., 2009; Sato and Stryker, 2010), but instead display limited OD plasticity with LTMD both during the critical period and as adults. Ubiquitin E3 ligase mutant mice also possess a deficit in the balance of excitatory and inhibitory cortical neurotransmission (Wallace et al., 2012). This phenotype is reminiscent of the mice mutant for GAD65 (above) in which the maturation of inhibitory cortical circuitry is impaired (Fagiolini and Hensch, 2000). Whether enhancing inhibition rescues visual plasticity in the UBE3A mice, akin to the effects of diazepam on GAD65 mutants, not been reported.

As both *FMR1* and *UBE3A* mutant mice display aberrant E/I balance, these associated deficits in experience-dependent visual plasticity may share a common circuit-level dysfunction. OD plasticity was evaluated in the both *FMR1* and *UBE3A* mutants with visually-evoked potentials (Dölen et al., 2007; Yashiro et al., 2009) and optical imaging of intrinsic signals (Sato and Stryker, 2010), techniques with less temporal and spatial specificity than either single-unit recordings or emerging approaches to study OD plasticity such as cell-attached recordings *in vivo* and calcium imaging (Kuhlman et al., 2013). As recent studies have begun to dissect with greater precision the interaction between components of the cortical circuitry that drive OD plasticity, this model may continue to improve as a useful framework for understanding if mutations in other genes also linked to syndromic forms of autism spectrum disorders, including neuroligin 3 (*NLGN3*), Src Homology-3 domain and multiple ankyrin repeat domains protein 3 (*SHANK3*), and Methyl CpG binding protein 2 (*MECP2*), interfere with experience-dependent plasticity conserved within neocortex.

## Directions of future vision research in mouse

A compendium of tools are now available for selectively expressing or deleting genes with various drivers of Cre recombinase (CRE), manipulating the activity specific neuronal populations with optogenetics, and measuring the activity of populations of neurons with genetically-encoded calcium indicators. These techniques are essential tools to dissect how experiences shape cortical circuitry. For example, by combining specific CRE drivers (Madisen et al., 2012) with CRE-dependent genetically-encoded calcium indicators, it may be possible to monitor plasticity during MD in specific cortical layers or subsets of interneurons with chronic calcium imaging *in vivo*. Similar experiments could then be performed on various mutant mice that lack OD plasticity in order to determine how and where plasticity is disrupted by these mutations, as well as within which neuronal populations these genes operate.

Importantly, the utility of the mouse is not restricted to OD plasticity. The mouse may serve as a model system for examining several outstanding questions in vision research. Several characteristics of visual circuitry are conserved between mouse and carnivores, including linear vs. nonlinear spatial summation, contrast-invariant tuning, and selectivity for stimulus parameters such as orientation and spatial frequency (Niell and Stryker, 2008). Thus, although mouse V1 lacks OD columns and possesses relatively poor spatial vision, it may nonetheless serve as a beneficial model system for investigating these properties of visual circuitry and potentially others, such as including disparity tuning (Scholl et al., 2013a) and/or simple and conserved relationships and connectivity between V1 and higher visual areas (Marshel et al., 2011). Overall, despite its small size and relatively simple architecture, the mouse visual system will continue to offer unique advantages for studying how experience shapes neural circuitry, allowing the field to ask—and answer—key questions with far-reaching relevance.

## Conflict of interest statement

The authors declare that the research was conducted in the absence of any commercial or financial relationships that could be construed as a potential conflict of interest.
